# Recursive Cluster Elimination (RCE) for classification and feature selection from gene expression data

**DOI:** 10.1186/1471-2105-8-144

**Published:** 2007-05-02

**Authors:** Malik Yousef, Segun Jung, Louise C Showe, Michael K Showe

**Affiliations:** 1 The Wistar Institute, Systems Biology Division, Philadelphia, PA 19104, USA

## Abstract

**Background:**

Classification studies using gene expression datasets are usually based on small numbers of samples and tens of thousands of genes. The selection of those genes that are important for distinguishing the different sample classes being compared, poses a challenging problem in high dimensional data analysis. We describe a new procedure for selecting significant genes as recursive cluster elimination (RCE) rather than recursive feature elimination (RFE). We have tested this algorithm on six datasets and compared its performance with that of two related classification procedures with RFE.

**Results:**

We have developed a novel method for selecting significant genes in comparative gene expression studies. This method, which we refer to as SVM-RCE, combines K-means, a clustering method, to identify correlated gene clusters, and Support Vector Machines (SVMs), a supervised machine learning classification method, to identify and score (rank) those gene clusters for the purpose of classification. K-means is used initially to group genes into clusters. Recursive cluster elimination (RCE) is then applied to iteratively remove those clusters of genes that contribute the least to the classification performance. SVM-RCE identifies the clusters of correlated genes that are most significantly differentially expressed between the sample classes. Utilization of gene clusters, rather than individual genes, enhances the supervised classification accuracy of the same data as compared to the accuracy when either SVM or Penalized Discriminant Analysis (PDA) with recursive feature elimination (SVM-RFE and PDA-RFE) are used to remove genes based on their individual discriminant weights.

**Conclusion:**

SVM-RCE provides improved classification accuracy with complex microarray data sets when it is compared to the classification accuracy of the same datasets using either SVM-RFE or PDA-RFE. SVM-RCE identifies clusters of correlated genes that when considered together provide greater insight into the structure of the microarray data. Clustering genes for classification appears to result in some concomitant clustering of samples into subgroups.

Our present implementation of SVM-RCE groups genes using the correlation metric. The success of the SVM-RCE method in classification suggests that gene interaction networks or other biologically relevant metrics that group genes based on functional parameters might also be useful.

## Background

The Matlab version of SVM-RCE can be downloaded from [1] under the "Tools->SVM-RCE" tab.

Classification of samples from gene expression datasets usually involves small numbers of samples and tens of thousands of genes. The problem of selecting those genes that are important for distinguishing the different sample classes being compared poses a challenging problem in high dimensional data analysis. A variety of methods to address these types of problems have been implemented [2-8]. These methods can be divided into two main categories: those that rely on filtering methods and those that are model-based or so-called wrapper approaches [2,4]. W. Pan [8] has reported a comparison of different filtering methods, highlighting similarities and differences between three main methods. The filtering methods, although faster than the wrapper approaches, are not particularly appropriate for establishing rankings among significant genes, as each gene is examined individually and correlations among the genes are not taken into account. Although wrapper methods appear to be more accurate, filtering methods are presently more frequently applied to data analysis than wrapper methods [4].

Recently, Li and Yang [9] compared the performance of Support Vector Machine (SVM) algorithms and Ridge Regression (RR) for classifying gene expression datasets and also examined the contribution of recursive procedures to the classification accuracy. Their study explicitly shows that the way in which the classifier penalizes redundant features in the recursive process has a strong influence on its success. They concluded that RR performed best in this comparison and further demonstrate the advantages of the wrapper method over filtering methods in these types of studies.

Guyon *et. al. *[10] compared the usefulness of RFE (for SVM) against the "naïve" ranking on a subset of genes. The naïve ranking is just the first iteration of RFE to obtain ranks for each gene. They found that SVM-RFE is superior to SVM without RFE and also to other multivariate linear discriminant methods, such as Linear Discriminant Analysis (LDA) and Mean-Squared-Error (MSE) with recursive feature elimination.

In this study, we describe a new method for gene selection and classification, which is comparable to or better than some methods which are currently applied. Our method (SVM-RCE) combines the K-means algorithm for gene clustering and the machine learning algorithm, SVMs [11], for classification and gene cluster ranking. The SVM-RCE method differs from related classification methods in that it first groups genes into correlated gene clusters by K-means and then evaluates the contributions of each of those clusters to the classification task by SVM. One can think of this approach as a search for those significant clusters of gene which have the most pronounced effect on enhancing the performance of the classifier. While we have used K-means and SVM to approach this problem, other combinations of clustering and classification methods could be used in approaching similar data analysis problems. Yu and Liu (2004) have discussed the redundancy and the relevance of features which is a related method [12].

Using SVM-RCE, the initial assessment of the performance of each individual gene cluster, as a separate feature, allows for the identification of those clusters that contribute the least to the classification. These are removed from the analysis while retaining those clusters which exhibit relatively better classification performance. We allow re-clustering of genes after each elimination step to allow the formation of new, potentially more informative clusters. The most informative gene clusters are retained for additional rounds of assessment until the clusters of genes with the best classification accuracy are identified (see Method section). Our results show that the classification accuracy with SVM-RCE is superior to SVM-RFE and PDA-RFE, which eliminate genes without explicit regard to their correlation with other genes.

Several recent studies [7,13,14] have also combined the K-means clustering algorithm and SVM but for very different purposes. In a previous study K-means was used to cluster the samples, rather than the features (genes). The sample clusters, represented as centroids, were then used as input to the SVM. In this case the sample clustering speeds the SVM learning by introducing fewer samples for training. Li *et. al. *[15] also used K-means in combination with SVM, but in this case K-means was used to cluster unlabelled sample data and SVM was used to develop the classifier among the clusters. However, none of the previous studies used K-means to cluster features and none are concerned with feature reduction, the principal aim of our method. Tang *et. al. *[16], proposed portioning the genes into clusters using the Fuzzy C-Means clustering algorithm. However, this study ranks each gene, in each individual cluster, by SVM coefficient weights rather than ranking each cluster as a unit. The size of the clusters, rather than the number of clusters, is reduced. A similar approach has recently been described by Ma and Huang [17] who propose a new method that takes into account the cluster structure, as described by correlation metrics, to perform gene selection at the cluster level and within-cluster gene level.

The following sections describe the individual components of the SVM-RCE algorithm. We present data showing the classification performance of SVM-RCE on complex data sets. We compare SVM-RCE with the performance of SVM-RFE and PDA-RFE and demonstrate the superior performance of SVM-RCE as measured by improved classification accuracy [18-20].

## Results

### Data used for assessment of classification accuracy

We tested the SVM-RCE method, described below, with several datasets. The preprocessed datasets for Leukemia and Prostate cancer were downloaded from the website [21] and used by the study [22]. The following is a brief description of these datasets.

#### • Leukemia

The leukemia dataset reported by Golub *et. al. *[23]. includes 72 patients to be classified into two disease types: Acute Lymphocytic Leukemia (ALL) and Acute Myeloid Leukemia (AML). 47 of the samples were from ALL patients (38 B-cell ALL and 9 T-cell ALL). An additional 25 cases were from patients with AML. Gene expression data was generated using the Affymetrix oligonucleotide microarrays with probe sets for 6,817 human genes. Data for 3571 genes remained, after preprocessing following the protocol described by Dudoit et. al. [24]. For simplicity we will refer to this data set as Leukemia(I). To properly compare the SVM-RCE performance with previous [9,25] studies, we split the data into two sets, a training set of 38 samples (27 ALL and 11 AML) and a test set of 34 samples (20 ALL and 14 AML) as in the original publication and used 7129 genes. The data was preprocessed by subtracting the mean and dividing the result by the standard deviation [9,23,25]. For simplicity, we will refer to this data as Leukemia (II).

#### • Prostate

This data set consists of 52 prostate tumor samples and 50 non-tumor prostate samples. It was generated using the Affymetrix platform with 9,000 genes. Data for 6033 genes remains after the preprocessing stage [22].

#### • CTCL Datasets (I) and (II)

Cutaneous T-cell lymphoma (CTCL) refers to a heterogeneous group of non-Hodgkin lymphomas of skin-homing T lymphocytes. CTCL(I) includes 18 patients and 12 controls [19] while CTCL(II) consist of 58 patients and 24 controls (Loboda et. al. unpublished). For more information about the data and preprocessing refer to [18,19].

#### • Head & neck vs. lung tumors (I)

Gene expression profiling was performed on a panel of 18 head and neck (HN) and 10 lung cancer (LC) tumor samples using Affymetrix U133A arrays. For further information refer to [26].

#### • Head & neck vs. lung tumors (II)

Gene expression profiling was performed on a panel of 52 patients with either primary lung (21 samples) or primary head and neck (31 samples) carcinomas, using the Affymetrix HG_U95Av2 high-density oligonucleotide microarray. For further information refer to Talbot *et. al. *[27].

The following two sections demonstrate the advantage of the SVM-RCE over SVM-RFE and PDA-RFE for selecting genes and accuracy of classification.

### Performance of SVM-RCE versus SVM-RFE and PDA-RFE

The three algorithms, SVM-RCE, PDA-RFE and SVM-RFE, were used to iteratively reduce the number of genes from the starting value in each dataset using intermediate classification accuracy as a metric.

We report the accuracy of SVM-RCE at the final 2 gene clusters, and two intermediate levels, usually 8 and 32 clusters, which correspond to 8 genes, 32 genes and 102 genes respectively. For SVM-RFE and PDA-RFE we report accuracy for comparable numbers of genes.

The relative accuracies of SVM-RCE, SVM-RFE and PDA-RFE are shown in Table 1. With the Leukemia(I) dataset, we observed an increased accuracy using SVM-RCE of 3% and 2% with ~12 and ~32 genes, respectively when compared to SVM-RFE. Comparable results with SVM-RFE required ~102 genes. The results obtained from the CTCL (I) analysis showed an improvement, using the SVM-RCE of about 11% and 6% with ~8 and ~32 genes, respectively, with a similar performance achieved with ~102 genes using SVM-RFE. The second CTCL data set (CTCL II, Loboda *et. al. *unpublished) showed an improvement using SVM-RCE of about 7%, 11% and 9% with ~8, ~34 and ~104 genes, respectively.

**Table 1:** Summary results for the SVM-RCE, SVM-RFE and PDA-RFE method. Summary results for the SVM-RCE, SVM-RFE and PDA-RFE method applied on 6 public datasets. #c field is the number of clusters for the SVM-RCE method. The #g field is the number of genes in the associated #c clusters for SVM-RCE, while for the SVM-RFE and PDA-RFE indicates the number of genes used.

	**Leukemia(I)**			**CTCL(I)**			**CTCL(II)**			**Head & Neck vs. Lung tumors (I)**			**Head & Neck vs. Lung tumors (II)**			**Prostate**		
	**#c**	**#g**	**ACC**	**#c**	**#g**	**ACC**	**#c**	**#g**	**ACC**	**#c**	**#g**	**ACC**	**#c**	**#g**	**ACC**	**#c**	**#g**	**ACC**

SVM-	2	12	99%	2	8	100%	2	8	91%	2	8	100%	2	9	100%	2	8	87%
RCE	3	32	98%	9	32	100%	9	34	96%	8	32	100%	6	32	100%	11	36	95%
	28	100	97%	32	101	100%	28	104	96%	28	103	100%	25	103	100%	32	100	93%

SVM-RFE		11	96%		9	89%		8	84%		8	92%		8	98%		8	93%
		32	96%		32	94%		32	85%		32	90%		32	98%		36	95%
		102	97%		102	100%		102	87%		102	90%		102	98%		102	94%

PDA-RFE		8	96%		8	92%		8	83%		8	89%		8	70%		8	94%
		32	96%		32	92%		33	81%		31	96%		32	98%		32	94%
		104	96%		104	95%		108	79%		109	96%		102	98%		104	90%

**Figure 1:**
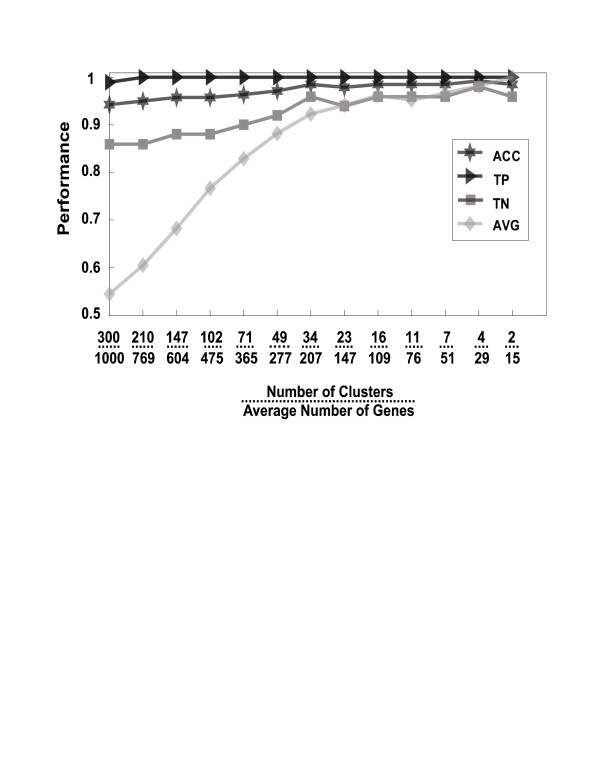
**Classification performance of SVM-RCE of Head & Neck vs. Lung tumors (I)**. All of the values are an average of 100 iterations of SVM-RCE. ACC is the accuracy, TP is the sensitivity, and TN is the specificity of the remaining genes determined on the test set. Avg is the average accuracy of the individual clusters at each level of clusters determined on the test set. The average accuracy increases as low-information clusters are eliminated. The x-axis shows the average number of genes hosted by the clusters.

**Figure 2:**
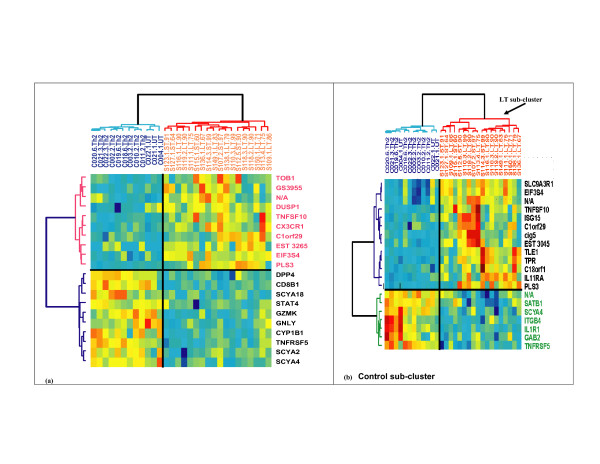
**Hierarchal cluster of CTCL(I) on the top 20 genes from SVM-RFE and SVM-RCE**. (a) Hierarchal cluster on the top 20 genes from SVM-RFE (b) Hierarchal cluster on the top 20 (~4 clusters) genes from SVM-RCE. Sample names that start with S are CTCL patients, while those that start with C are for controls. LT = long term, ST = short term.

We also compared results for two additional datasets: Head and Neck Squamous Cell carcinoma (HNSCC) and Lung Squamous Cell carcinoma (LSCC) [26] (*Head & Neck vs. Lung tumors (I)*). SVM-RCE shows an increase in accuracy over SVM-RFE of 8%, 10% and 10% with ~8, ~32, and ~103 genes, respectively. A similar dataset comparing HNSCC and LSCC [27] (*Head & Neck vs. Lung tumors (II)*) was also subjected to both methods and a ~2% increase was observed, with the SVM-RCE, using ~8, ~32, and ~102 of genes (100% SVM-RCE and 98% SVM-RFE). The Prostate cancer dataset yielded better accuracy using SVM-RFE with ~8 genes (an increase of about 6% over SVM-RCE), but similar performances were found at higher gene numbers. The same superiority of SVM-RCE is observed when comparing the SVM-RCE with PDA-RFE. These results are also shown in Table 1. Figures 1 and 2 (Additional Material File 1: Hierarchical clustering and Multidimensional scaling (MDS) of the top genes detected by SVM-RCE and SVM-RFE) use hierarchal clustering and multidimensional scaling (MDS) [28] to help illustrate the improved classification accuracy of SVM-RCE for two of the data sets, Head&Neck(I) and CTCL(I). The genes selected by SVM-RCE clearly separate the two classes while the genes selected by SVM-RFE place one or two samples on the wrong side of the separating margin.

### Comparison with Li and Yang study

Recently, Li and Yang [9] conducted a study comparing SVM and Ridge Regression(RR) to understand the success of RFE and to determine how the classification accuracy depends on the specific classification algorithm that is chosen. They found that RR applied on the Leukemia(II) dataset has zero errors, with only 3 genes, while SVM [25] only attained the same zero errors with 8 genes. We compared these studies to our results, using SVM-RCE (*n *= 100, *m *= 2, *d *= 0.1, *n_g *= 500), where 1 error was found with 3 genes (KRT16, SELENBP1 and SUMO1) and zero errors with 7 genes. The one misclassified sample is located at the margin, between the two classes.

### Tuning and parameters

We have also examined the effect of using more genes (more than 300) selected by t-test from the training set as input for SVM-RCE (See section "Choice of Parameters" for more details). While no dramatic changes are observed, there is some small degradation in the performance (1–2%) as progressively more genes are input. A similar observation has been reported when SVM-RFE is applied to proteomic datasets by Rajapakse *et. al. *[29].

For demonstrating the convergence of the algorithm to the optimal solution and to give a more visual illustration of the SVM-RCE method, we have tracked the mean performance over all the clusters for each reduction level. Figure 1 illustrates the performance on SVM-RCE for the *Head & Neck vs. Lung tumors (I) *dataset over different levels of clusters. The analysis starts with 1000 genes selected by t-test from the training set that were distributed into 300 clusters (*n *= 300, *m *= 2, *d *= 0.3, *n_g *= 1000) and then recursively decreased to 2 clusters. The mean classification performance on the test set per cluster at each level of reduction (Figure 1 line AVG) is dramatically improved from ~55% to ~95% as the number of clusters decreases supporting the suggestion that less-significant clusters are being removed while informative clusters are retained as RCE is employed.

### Speed and stability

The execution time for our SVM-RCE MATLAB code is greater than PDA-RFE or SVM-RFE, which use the C programming language. For example, applying the SVM-RCE on a Personal Computer with P4-Duo-core 3.0 GHz and 2GB RAM on *Head & Neck vs. Lung tumors (I) *took approximately 9 hours for 100 iterations (10-folds repeated 10 times), while SVM-RFE (with the svm-gist package) took 4 minutes.

To estimate the stability of the results, we have re-run SVM-RCE while tracking the performance at each iteration, over each level of gene clusters. The mean accuracy and the standard deviation (stdv) are calculated at the end of the run. The *Head & Neck vs. Lung tumors (I) *data set with SVM-RCE has a stdv of 0.04–0.07. Surprisingly, SVM-RFE with the same data yields a stdv range of 0.2–0.23. A similar stdv range (0.17–0.21) was returned when SVM-RFE was re-employed with 1000 iterations. Therefore, SVM-RCE is more robust and more stable than SVM-RFE.

K-means is sensitive to the choice of the seed clusters, but clustering results should converge to a local optimum on repetition. For stability estimations, we have carried out SVM-RCE (on *Head & Neck vs. Lung tumors (I)) *with values of *u *of 1, 10, and 100 repetitions (see sub-section K-means Cluster), and compared the most informative 20 genes returned from each experiment. ~80% of the genes are common to the three runs, which suggests that the SVM-RCE results are robust and stable. Moreover, similar accuracy was obtained from each experiment.

### Is there an advantage, besides increased accuracy, to using SVM-RCE for gene selection?

Our results suggest that SVM-RCE can reveal important information that is not captured by methods that assess the contributions of each gene individually. Although we have limited our initial observations, for simplicity, to the top 2 clusters needed for separation of datasets with 2 known classes of samples, one can expand the analysis to capture, for example, the top 4 clusters of genes. We hypothesized that by increasing the number of clusters selected that we might be able to identify sample sub-clusters, which may be present in a specific dataset. The CTCL(I) dataset illustrates this possibility. Figure 2 shows the hierarchical clusters generated using the top 4 significant clusters (about 20 genes) revealed by SVM-RCE (Figure 2(b)) and (Figure 2(a)) with comparable numbers of genes (20 genes) identified by SVM-RFE. The 4 clusters of genes in Figure 2(b) (two up-regulated in patients and another two down-regulated) appear to identify sub-clusters of samples present in each class. For example, we see that four samples from the control class (C021.3.Th2, C020.6.Th2, CO04.1.UT and C019.6.Th2) form a sub-cluster identified by the genes TNFRSF5, GAB2, IL1R1 and ITGB4 (See Figure 2(b) "Control sub-cluster" label). Three of these 4 controls represent a control class (Th2) that has been treated with IL-4. In addition, a sub-cluster of genes up-regulated in patients (SLC9A3R1 through cig5) cluster 9 patients distinguished as long-term survivors (LT) and 1 short-term (ST) survivor from the remaining patients (See Figure 2(b) "LT sub-cluster" label). However, no specific sub-pattern is apparent in Figure 2(a) using the top 20 genes obtained from SVM-RFE. See "Additional Material File 2: Comparison of the CTCL(I) genes selected by SVM-RCE and SVM-RFE and concomitant clustering of genes and samples", which shows additional structure of the data obtained in the classifications with gene clusters obtained using SVM-RCE compared with SVM-RFE. This structure arises because SVM-RCE selects different genes for the classification.

## Conclusion

In this paper we present a novel method SVM-RCE for selecting significant genes for (supervised) classification of microarray data, which combines the K-means clustering method and SVM classification method. SVM-RCE demonstrated improved (or equivalent in one case) accuracy compared to SVM-RFE and PDA-RFE on 6 microarray datasets tested.

Defining the minimum number of clusters required for accurate classification can be a challenging task. With our approach, the number of clusters and cluster size is determined arbitrarily at the onset of the analysis by the investigator and, as the algorithm proceeds, the least informative clusters are progressively removed. However, in order to avoid producing redundant clusters, we believe that this step needs to be automated to obtain an optimum final value. A number of statistical techniques [30,31] have been developed to estimate this number.

The RFE procedure associated with the SVM (or PDA) is designed to estimate the contributions of individual genes to the classification task, whereas the RCE procedure, associated with SVM-RCE, is designed to estimate the contribution of a cluster of genes for the classification task. Other studies [32-36] have used biological knowledge-driven approaches for assessment of the generated gene clusters by unsupervised methods. Our method provides the top *n *clusters required to most accurately differentiate the two pre-defined classes.

The relationship between the genes of a single cluster and their functional annotation is still not clear. Clare and Kind [37] found in yeast, that clustered genes do to not have correlated functions as might have been expected. One of the merits of the SVM-RCE is its ability to group the genes using different metrics. In the present study, the statistical correlation metric was used. However, one could use biological metrics such as gene interaction network information or functional annotation for clustering genes (*Cluster step *in the SVM-RCE algorithm) to be examined with the SVM-RCE for their contribution to the classification task [38]. In this way, the outcome would be a set of significant genes that share biological networks or functions.

The results presented suggest that the selection of significant genes for classification, using SVM-RCE, is more reliable than the SVM-RFE or PDA-RFE. SVM-RFE uses the weight coefficient, which appears in the SVM formula, to indicate the contribution of each gene to the classifier. However, the exact relation between the weights and performance is not well understood. One could argue that some genes with low absolute weights are important and their low ranking is a result of other dominant correlated genes. The success of SVM-RCE suggests that estimates based on the contribution of genes, which share a similar profile (correlated genes), is important and gives each group of genes the potential to be ranked as a group. Moreover, the genes selected by SVM-RCE are guaranteed to be useful to the overall classification since the measurement of retaining or removing genes (cluster of genes) is based on their contribution to the performance of the classifier as expressed by the *Score *(·) measurement. Similarly Tang *et. al*. [16] has shown that partitioning the genes into clusters, followed by performing estimates of the ranks of each gene by SVM, generates improved results compared to the traditional SVM-RFE. Ma and Huang [17] have also shown improved results when feature selection takes account of the structure of the genes clusters. These results suggest that clustering the genes and performing an estimation of individual gene clusters is the key to enhance the performance and improve the grouping of significant genes. The unsupervised clustering used by SVM-RCE has the additional possibility of identifying biologically or clinically important sub-clusters of samples.

## Methods

The following sub-sections describe our method and its main components. SVM-RCE combines K-means, a clustering method, to identify correlated gene clusters, and Support Vector Machines (SVMs), a supervised machine learning classification method, to identify and score (rank) those gene clusters for accuracy of classification. K-means is used initially to group genes into clusters. After scoring by SVM the lowest scoring clusters are removed. The remaining clusters are merged, and the process is repeated.

### The SVM-RCE method-scoring gene clusters

We assume that given dataset D with S genes. The data partitioned into two parts, one for training (90% of the samples) and the other (10% of the samples) for testing.

Let *X *denote a two-class training dataset that consisting of ℓ samples and *S *genes. We define a score measurement for any list *S *of genes as the ability to differentiate the two classes of samples by applying linear SVM. To calculate this score we carry out a random partition the training set *X *of samples into *f *non-overlapping subsets of equal sizes (*f*-folds). Linear SVM is trained over *f*-1 subsets and the remaining subset is used to calculate the performance. This procedure is repeated *r *times to take into account different possible partitioning. We define *Score*(*X*(*S*), *f*, *r*) as the average accuracy of the linear SVM over the data *X *represented by the S genes computed as *f*-folds cross validation repeated *r *times. We set *f *to 3 and *r *to 5 as default values. Moreover, if the *S *genes are clustered into sub-clusters of genes *S*_1_, *S*_2_,..., *S*_*n *_then we define the *Score*(*X*(*s*_*i*_), *f*, *r*) for each sub-cluster while *X*(*s*_*i*_) is the data *X *represented by the genes of *S*_*i*_.

**Figure 3:**
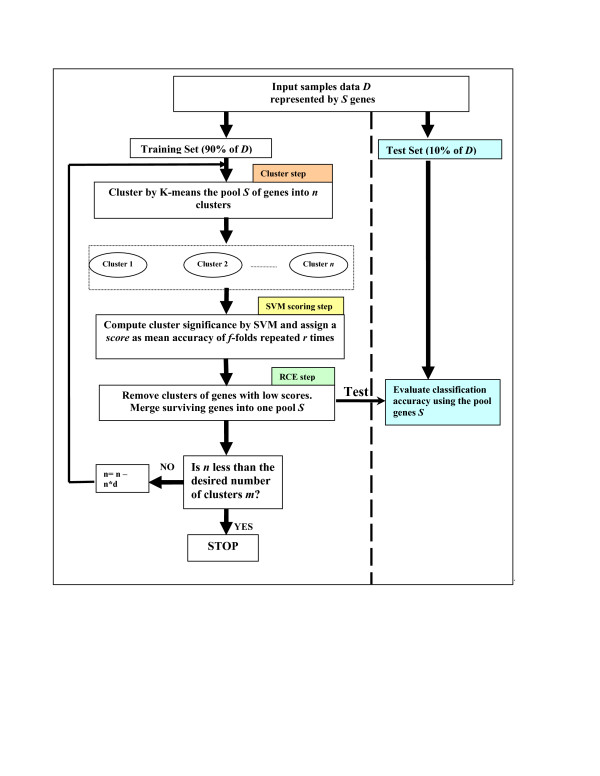
**The description of the SVM-RCE algorithm**. A flowchart of the SVM-RCE algorithm consists of main three steps: the *Cluster step *for clustering the genes, the *SVM scoring step *for assessment of significant clusters and the *RCE step *to remove clusters with low score

The central algorithm of SVM-RCE method is described as a flowchart in Figure 3. It consists of three main steps applied on the training part of the data: the *Cluster step *for clustering the genes, the *SVM scoring step *for computing the *Score*(*X*(*s*_*i*_), *f*, *r*) of each cluster of genes and the *RCE step *to remove clusters with low score, as follows:

### Algorithm SVM-RCE (input data *D*)

*X *= the training dataset

*S *= genes list (all the genes) or top *n_g *genes by t-test

*n *= initial number of clusters

*m *= final number of clusters

*d *= the reduction parameter

While (*n *≤ *m*) do

1. Cluster the given genes *S *into *n *clusters *S*_1_, *S*_2_,..., *S*_*n *_using K-means (**Cluster step**)

2. For each cluster *i *= 1..*n *calculate its *Score*(*X*(*s*_*i*_), *f*, *r*) (**SVM scoring step)**

3. Remove the *d% *clusters with lowest score (**RCE step**)

4. Merge surviving genes again into one pool *S*

5. Decrease *n *by *d%*.

The basic approach of the SVM-RCE is to first cluster the gene expression profiles into *n *clusters, using K-means. A score *Score*(*X*(*s*_*i*_), *f*, *r*), is assigned to each of the clusters by linear SVM, indicating its success at separating samples in the classification task. The *d% *clusters (or *d *clusters) with the lowest scores are then removed from the analysis. Steps 1 to Step 5 are repeated until the number *n *of clusters is decreased to *m*.

Let *Z *denote the testing dataset. At step 4 an SVM classifier is built from the training dataset using the surviving genes S. This classifier is then tested on *Z *to estimate the performance. See Figure 3 the "Test" panel on the right side.

For the current version, the choice of *n *and *m *are determined by the investigator. In this implementation, the default value of *m *is 2, indicating that the method is required to capture the top 2 significant clusters (groups) of genes. However, accuracy is determined after each round of cluster elimination and a higher number of clusters could be more accurate than the final two.

Various methods have been used for classification studies to find the optimal subset of genes that gives the highest accuracy [39] in distinguishing members of different sample classes. With SVM-RCE, one can think of this process as a search in the gene-clusters space for the *m *clusters, of correlated genes, that give the highest classification accuracy. In the simplest case, the search is reduced to the identification of one or two clusters that define the class differences. These might include the important up-regulated and the important down-regulated genes. While SVM-RCE and SVM-RFE are related, in that they both assess the relative contributions of the genes to the classifier, SVM-RCE assesses the contributions of groups of correlated genes instead of individual genes (SVM scoring step in Figure 3). Additionally, although both methods remove the least important genes at each step, SVM-RCE scores and removes clusters of genes, while SVM-RFE scores and removes a single or small numbers of genes at each round of the algorithm.

### Implementation

The gist-svm [40] package was used for the implementation of SVM-RFE, with linear kernel function (dot product), with default parameters. In gist-svm the SVM employs a two-norm soft margin with C = 1 as penalty parameter. See [41] for more details.

SVM-RCE is coded in MATLAB while the Bioinformatics Toolbox 2.1 release is used for the implementation of linear SVM with two-norm soft margin with *C *= 1 as penalty parameter. The core of PDA-RFE is implemented in C programming language in our lab (Showe Laboratory, The Wistar Institute) with a JAVA user interface (Manuscript in preparation). We haven't used any tuning parameter procedure for optimization.

### Choice of parameters

In order to ensure a fair comparison and to decrease the computation time, we started with the top 300 (*n_g *= 300) genes selected by t-test from the training set for all methods. However, as was observed by Rajapakse *et. al.*(2005) [29], using t-statistics for reducing the number of onset genes subjected to SVM-RFE is not only efficient, but it also enhances the performance of the classifier. However, a larger initial starting set might result in biologically more informative clusters.

For all of the results presented, 10% (*d *= 0.1) is used for the gene cluster reduction for SVM-RCE and 10% of the genes with SVM-RFE and PDA-RFE. For SVM-RCE, we started with 100 (*n *= 100) clusters and stopped when 2 (*m *= 2) clusters remained. 3-fold (*f *= 3) repeated 5 (*r *= 5) times was used in the SVM-RCE method to evaluate the score of each cluster (SVM scoring step in Figure 3). It is obvious that one can use more stringent evaluation parameters, by increasing the number of repeated cross-validations, at the price of increasing the computational time. In some difficult classification cases, it is worth doing this in order to enhance the prediction accuracy.

### Evaluation

For evaluating the over-all performance of SVM-RCE and SVM-RFE (and PDA-RFE), 10-fold cross validation (9 fold for training and 1 fold for testing), repeated 10 times, was employed. After each round of feature or cluster reduction, the accuracy was calculated on the hold-out test set. For each sample in the test set, a score assigned by SVM indicates its distance from the discriminate hyper-plane generated from the training samples, where a positive value indicates membership in the positive class and a negative value indicates membership in the negative class. The class label for each test sample is determined by averaging all 10 of its SVM scores and it is based on this value that the sample is classified. This method for calculating the accuracy gives a more accurate measure of the performance, since it captures not only whether a specific sample is positively (+1) or negatively (-1) classified, but how well it is classified into each category, as determined by a score assigned to each individual sample. The score serves as a measure of classification confidence. The range of scores provides a confidence interval.

### K-means cluster

Clustering methods are unsupervised techniques where the labels of the samples are not assigned. K-means [42] is a widely used clustering algorithm. It is an iterative method that groups genes with correlated expression profiles into *k *mutually exclusive clusters. *k *is a parameter that needs to be determined at the onset. The starting point of the K-means algorithm is to initiate *k *randomly generated seed clusters. Each gene profile is associated with the cluster with the minimum distance (different metrics could be used to define distance) to its 'centroid'. The centroid of each cluster is then recomputed as the average of all the cluster gene members' profiles. The procedure is repeated until no changes in the centroids, for the various clusters, are detected. Finally, this algorithm aims at minimizing an *objective function *with *k *clusters:

F(date;k)=∑j=1k∑i=1t‖gij−cj‖2 where t is number of genes.
 MathType@MTEF@5@5@+=feaafiart1ev1aaatCvAUfKttLearuWrP9MDH5MBPbIqV92AaeXatLxBI9gBaebbnrfifHhDYfgasaacH8akY=wiFfYdH8Gipec8Eeeu0xXdbba9frFj0=OqFfea0dXdd9vqai=hGuQ8kuc9pgc9s8qqaq=dirpe0xb9q8qiLsFr0=vr0=vr0dc8meaabaqaciaacaGaaeqabaqabeGadaaakeaacqWGgbGrcqGGOaakcqWGKbazcqWGHbqycqWG0baDcqWGLbqzcqGG7aWocqWGRbWAcqGGPaqkcqGH9aqpdaaeWbqaamaaqahabaWaauWaaeaacqWGNbWzdaqhaaWcbaGaemyAaKgabaGaemOAaOgaaOGaeyOeI0Iaem4yam2aaSbaaSqaaiabdQgaQbqabaaakiaawMa7caGLkWoadaahaaWcbeqaaiabikdaYaaaaeaacqWGPbqAcqGH9aqpcqaIXaqmaeaacqWG0baDa0GaeyyeIuoaaSqaaiabdQgaQjabg2da9iabigdaXaqaaiabdUgaRbqdcqGHris5aOGaeeiiaaIaee4DaCNaeeiAaGMaeeyzauMaeeOCaiNaeeyzauMaeeiiaaIaemiDaqNaeeiiaaIaeeyAaKMaee4CamNaeeiiaaIaeeOBa4MaeeyDauNaeeyBa0MaeeOyaiMaeeyzauMaeeOCaiNaeeiiaaIaee4Ba8MaeeOzayMaeeiiaaIaee4zaCMaeeyzauMaeeOBa4MaeeyzauMaee4CamNaeiOla4caaa@74E1@

where || ||^2 ^is the distance measurement between gene *g*_*i *_profile and the cluster centroid *c*_*j*_. The "correlation" distance measurement was used as a metric for the SVM-RCE approach. The correlation distance between genes *g*_*r *_and *g*_*s *_is defined as:

drs=1−(gr−g¯r)(gs−g¯s)′(gr−g¯r)(gr−g¯r)′(gs−g¯s)(gs−g¯s)′ where g¯r=1t∑jgrj and g¯s=1t∑jgsj
 MathType@MTEF@5@5@+=feaafiart1ev1aaatCvAUfKttLearuWrP9MDH5MBPbIqV92AaeXatLxBI9gBaebbnrfifHhDYfgasaacH8akY=wiFfYdH8Gipec8Eeeu0xXdbba9frFj0=OqFfea0dXdd9vqai=hGuQ8kuc9pgc9s8qqaq=dirpe0xb9q8qiLsFr0=vr0=vr0dc8meaabaqaciaacaGaaeqabaqabeGadaaakeaacqWGKbazdaWgaaWcbaGaemOCaiNaem4Camhabeaakiabg2da9iabigdaXiabgkHiTmaalaaabaGaeiikaGIaem4zaC2aaSbaaSqaaiabdkhaYbqabaGccqGHsislcuWGNbWzgaqeamaaBaaaleaacqWGYbGCaeqaaOGaeiykaKIaeiikaGIaem4zaC2aaSbaaSqaaiabdohaZbqabaGccqGHsislcuWGNbWzgaqeamaaBaaaleaacqWGZbWCaeqaaOGafiykaKIbauaaaeaadaGcaaqaaiabcIcaOiabdEgaNnaaBaaaleaacqWGYbGCaeqaaOGaeyOeI0Iafm4zaCMbaebadaWgaaWcbaGaemOCaihabeaakiabcMcaPiabcIcaOiabdEgaNnaaBaaaleaacqWGYbGCaeqaaOGaeyOeI0Iafm4zaCMbaebadaWgaaWcbaGaemOCaihabeaakiqbcMcaPyaafaaaleqaaOWaaOaaaeaacqGGOaakcqWGNbWzdaWgaaWcbaGaem4CamhabeaakiabgkHiTiqbdEgaNzaaraWaaSbaaSqaaiabdohaZbqabaGccqGGPaqkcqGGOaakcqWGNbWzdaWgaaWcbaGaem4CamhabeaakiabgkHiTiqbdEgaNzaaraWaaSbaaSqaaiabdohaZbqabaGccuGGPaqkgaqbaaWcbeaaaaGccqqGGaaicqqG3bWDcqqGObaAcqqGLbqzcqqGYbGCcqqGLbqzcqqGGaaicuWGNbWzgaqeamaaBaaaleaacqWGYbGCaeqaaOGaeyypa0ZaaSaaaeaacqaIXaqmaeaacqWG0baDaaWaaabuaeaacqWGNbWzdaWgaaWcbaGaemOCaiNaemOAaOgabeaaaeaacqWGQbGAaeqaniabggHiLdGccqqGGaaicqqGHbqycqqGUbGBcqqGKbazcqqGGaaicuWGNbWzgaqeamaaBaaaleaacqWGZbWCaeqaaOGaeyypa0ZaaSaaaeaacqaIXaqmaeaacqWG0baDaaWaaabuaeaacqWGNbWzdaWgaaWcbaGaem4CamNaemOAaOgabeaaaeaacqWGQbGAaeqaniabggHiLdaaaa@9312@

K-means is sensitive to the choice of the seed clusters (initial centroids) and different methods for choosing the seed clusters can be considered. At the K-means step (Cluster step in Figure 3) of SVM-RCE, *k *genes are *randomly *selected to form the seed clusters and this process is repeated several times (*u *times) in order to reach the optimal, with the lowest value of the objective function *F*(*data*; *k*).

Clustering methods are widely used techniques for microarray data analysis. Gasch and Eisen [43] used a heuristically modified version of Fuzzy K-means clustering to identify overlapping clusters and a comparison with the standard K-means method was reported. Monti *et. al. *[44] report a new methodology of class discovery, based on clustering methods, and present an approach for validation of clustering and assess the stability of the discovered clusters.

### Support Vector Machines (SVMs)

Support Vector Machines (SVMs) is a learning machine developed by Vapnik [11]. The performance of this algorithm, as compared to other algorithms, has proven to be particularly useful for the analysis of various classification problems, and has recently been widely used in the bioinformatics field [45-47]. Linear SVMs are usually defined as SVMs with linear kernel. The training data for linear SVMs could be linear non-separable and then soft-margin SVM could be applied. Linear SVM separates the two classes in the training data by producing the optimal separating hyper-plane with a maximal margin between the class 1 and class 2 samples. Given a training set of labeled examples(*x*_*i*_, *y*_*i*_), *i *= 1,..., ℓ where *x*_*i *_∈ *R' *and *y*_*i *_∈ {+1, -1}, the support vector machines (SVMs) find the separating hyper-plane of the form *w*·*x*+*b *= 0 *w *∈ *R'*, *b *∈ *R*. Here, *w *is the "normal" of the hyper-plane. The constant *b *defines the position of the hyper-plane in the space. One could use the following formula as a predictor for a new instance: *f*(*x*) = *sign*(*w*·*x *+ *b*) for more information see Vapnik [11].

The application of SVMs to gene expression datasets can be divided into two basic problems: one for gene function discovery and the other for classification. As an example of the first category, Brown, Grundy *et. al. *[48] successfully used SVM for the "*identification of biological functionally related genes*", where essentially two group of genes are identified. One group consists of genes that have a common function and the other group consists of genes that are not members of that functional class. Comparisons with several SVMs, that use different similarity metrics, were also conducted. SVMs performance was reported to be superior to other supervised learning methods for functional classification. Similarly, Eisen, Spellman *et. al*. [49] used a clustering method with Pearson correlation, as a metric, in order to capture genes with similar expression profiles.

As an example of the second category, Furey *et. al. *[50] used SVM for the classification of different samples into classes and as a statistical test for gene selection (filter approach).

### SVM Recursive Feature Elimination (SVM-RFE)

SVM-RFE [25] is a SVM based model that removes genes, recursively based on their contribution to the discrimination, between the two classes being analyzed. The lowest scoring genes by coefficient weights are removed and the remaining genes are scored again and the procedure is repeated until only a few genes remain. This method has been used in several studies to perform classification and gene selection tasks [9,51].

Furlanello *et. al*. [51] developed an entropy recursive feature elimination (E-RFE) in order to accelerate (100×) the RFE step with the SVM. However, they do not demonstrate any improvement in the classification performance compared to the regular SVM-RFE approach. Several other papers, as in Kai-Bo *et. al. *[6], propose a new technique that relies on a backward elimination procedure, which is similar to SVM-RFE. They suggest that their method is selecting better sub-sets of genes and that the performance is enhanced compared to SVM-RFE. Huang *et. al. *[52] explore the influence of the penalty parameter *C *on the performance of SVM-RFE, finding that one dataset *C *could be better classified when *C *was optimized.

In general, choosing appropriate values of the algorithm parameters (penalty parameter, kernel-function, etc) can often influence performance. Recently, Zhang *et. al. *[5] proposed R-SVM as a recursive support vector machine algorithm to select important features in SELDI data. The R-SVM was compared to the SVM-RFE and is suggested to be more robust to noise. No improvement in the classification performance was found.

## Competing interests

The author(s) declare that they have no competing interests.

## Availability and requirements

The Matlab version of SVM-RCE can be downloaded from [1] under the "Tools->SVM-RCE" tab.

## Authors' contributions

Malik Yousef, Louise C. Showe and Michael K. Showe equally contributed to the manuscript while Segun Jung revised the Matlab code, which was written by Malik Yousef, to make it available over the web, obtained the PDA-RFE results, and measured the statistical significance of the method. All authors approved the manuscript.

## Supplementary Material

Additional File 1 **Hierarchical clustering and Multidimensional scaling (MDS) of the top genes detected by SVM-RCE and SVM-RFE**. helps illustrate the improved classification accuracy of SVM-RCE for two of the data sets, Head&Neck(I) and CTCL(I). Click here for file

Additional File 2 **Comparison of the CTCL(I) genes selected by SVM-RCE and SVM-RFE and concomitant clustering of genes and samples**. shows additional structure of the data obtained in the classifications with gene clusters obtained using SVM-RCE compared with SVM-RFE. Click here for file
