# Supplementary material for: Recursive Cluster Elimination (RCE) for classification and feature selection from gene expression data
Source: BMC Bioinformatics. 2007 May 2;8:144. doi: 10.1186/1471-2105-8-144 (PMC1877816; doi:10.1186/1471-2105-8-144)
Supplement: Additional File 1 — Hierarchical clustering and Multidimensional scaling (MDS) of the top genes detected by SVM-RCE and SVM-RFE. helps illustrate the improved classification accuracy of SVM-RCE for two of the data sets, Head&Neck(I) and CTCL(I). [file 1471-2105-8-144-S1.pdf]

File 1: Hierarchical clustering and Multidimensional scaling (MDS) of the top genes detected by SVM- RCE and SVM-RFE

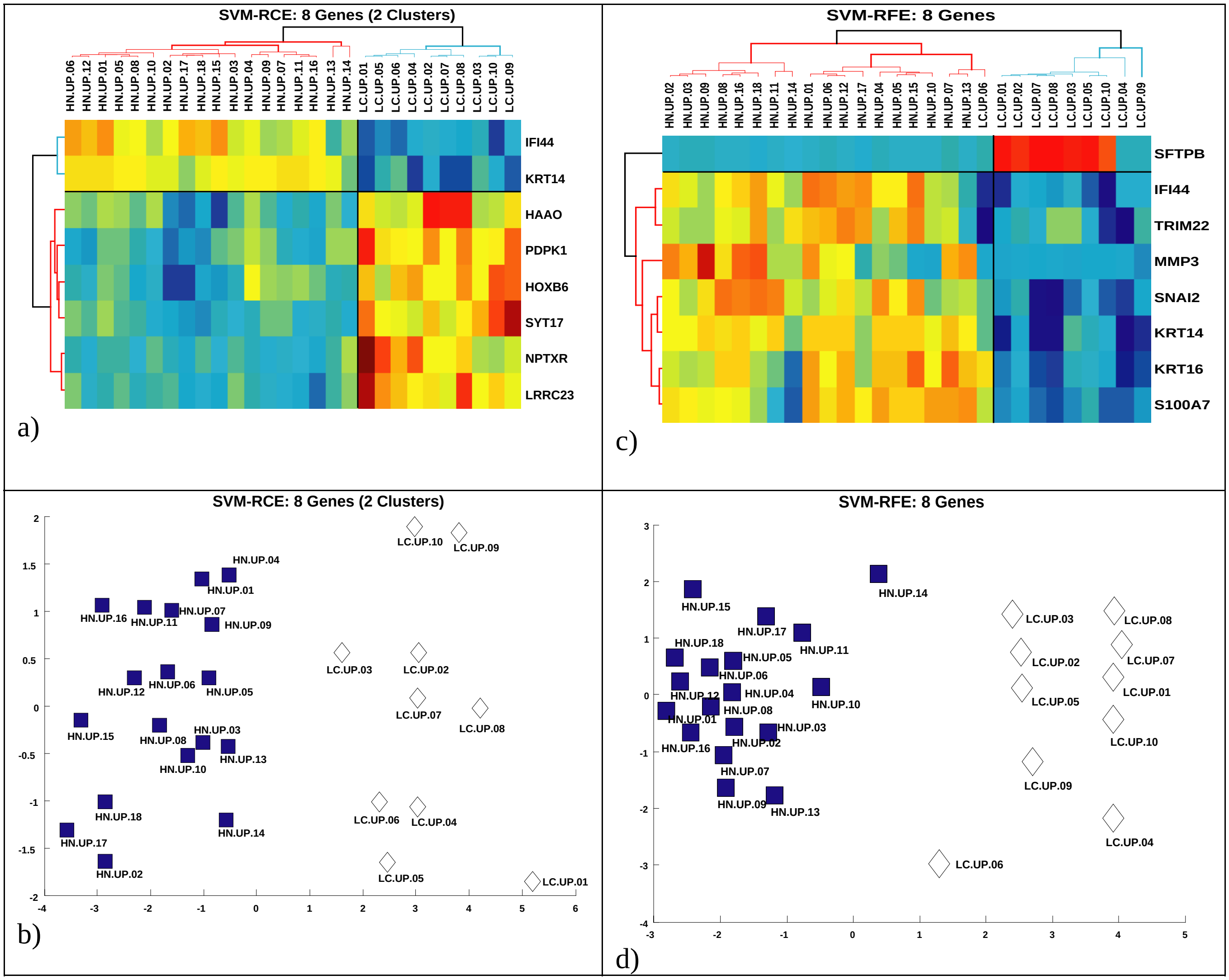

Figure 1: The results of SVM-RCE and SVM-RFE on the Head&Neck(I) dataset. The left panel is the Hierarchical cluster (a) and Multidimensional scaling (MDS) (b) of the top two clusters (8 genes) detected by SVM-RCE while the right panel is the hierarchical cluster (c) and Multidimensional scaling (MDS) (d) of the top 8 genes detected by SVM-RFE. Sample names that start with HN are Head&neck tumor samples while those that start with LC are lung cancer (LC) tumor samples.

The RCE procedure gives perfect classification while the RFE procedure misclassifies one sample (LCUP06).

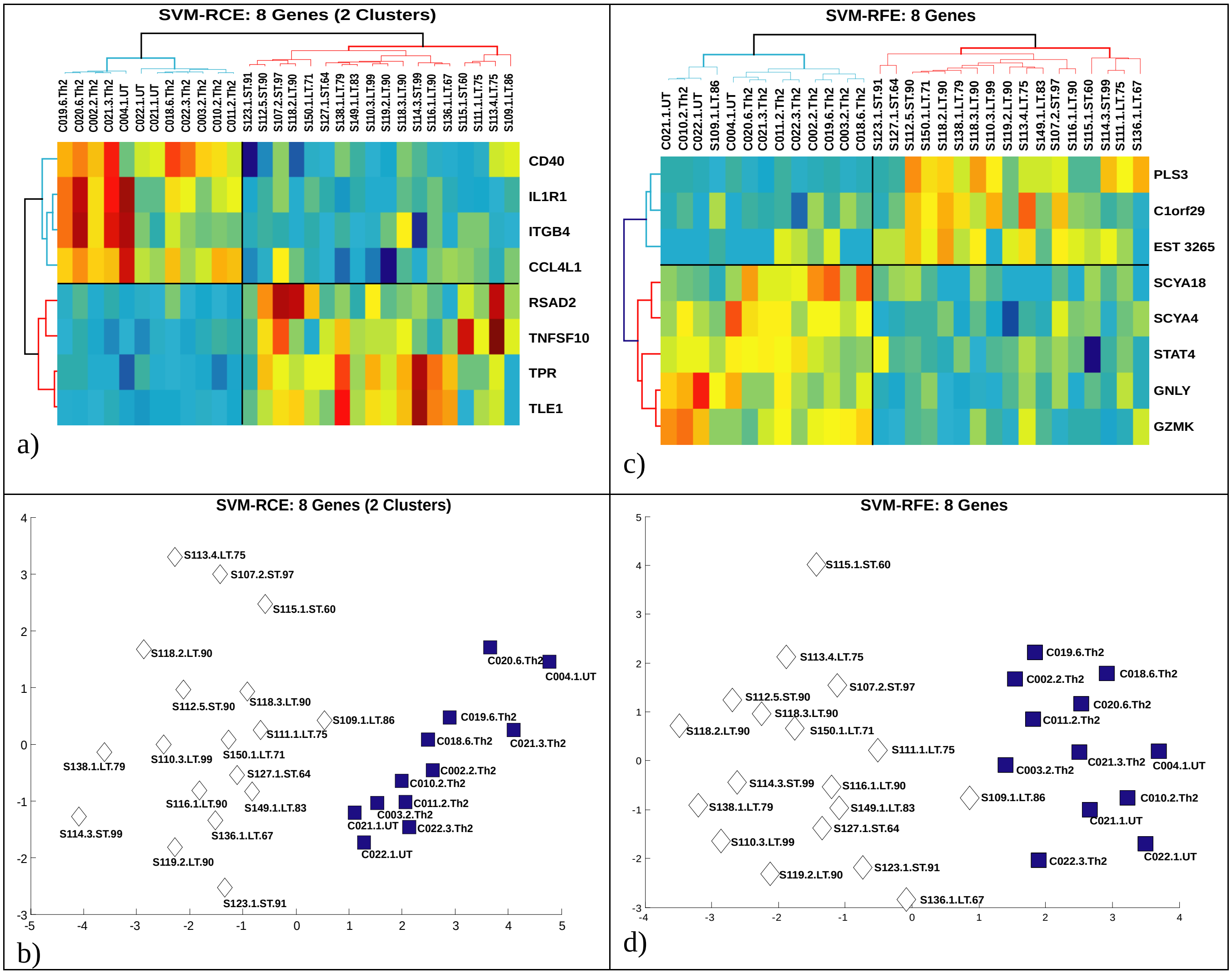

Figure 2: The results of SVM-RCE and SVM-RFE on the CTCL(I) dataset. The left panel is the hierarchical cluster (a) and Multidimensional scaling (MDS) (b) of the top two clusters (8 genes) detected by SVM-RCE while the right panel is the hierarchal cluster (c) and Multidimensional scaling (MDS) (d) of the top 8 genes detected by SVM-RFE. Sample names that start with S are CTCL patients, while those that start with C are for controls. LT= long term survivor, ST=short term survivor. The RCE procedure perfectly separates patients from controls, while the genes selected by RFE misclassify patient S109 as a control.
