# Supplementary material for: Recursive Cluster Elimination (RCE) for classification and feature selection from gene expression data
Source: BMC Bioinformatics. 2007 May 2;8:144. doi: 10.1186/1471-2105-8-144 (PMC1877816; doi:10.1186/1471-2105-8-144)
Supplement: Additional File 2 — Comparison of the CTCL(I) genes selected by SVM-RCE and SVM-RFE and concomitant clustering of genes and samples. shows additional structure of the data obtained in the classifications with gene clusters obtained using SVM-RCE compared with SVM-RFE. [file 1471-2105-8-144-S2.pdf]

File 2: Comparison of the CTCL(I) genes selected by SVM-RCE and SVM-RFE and concomitant clustering of genes and samples.

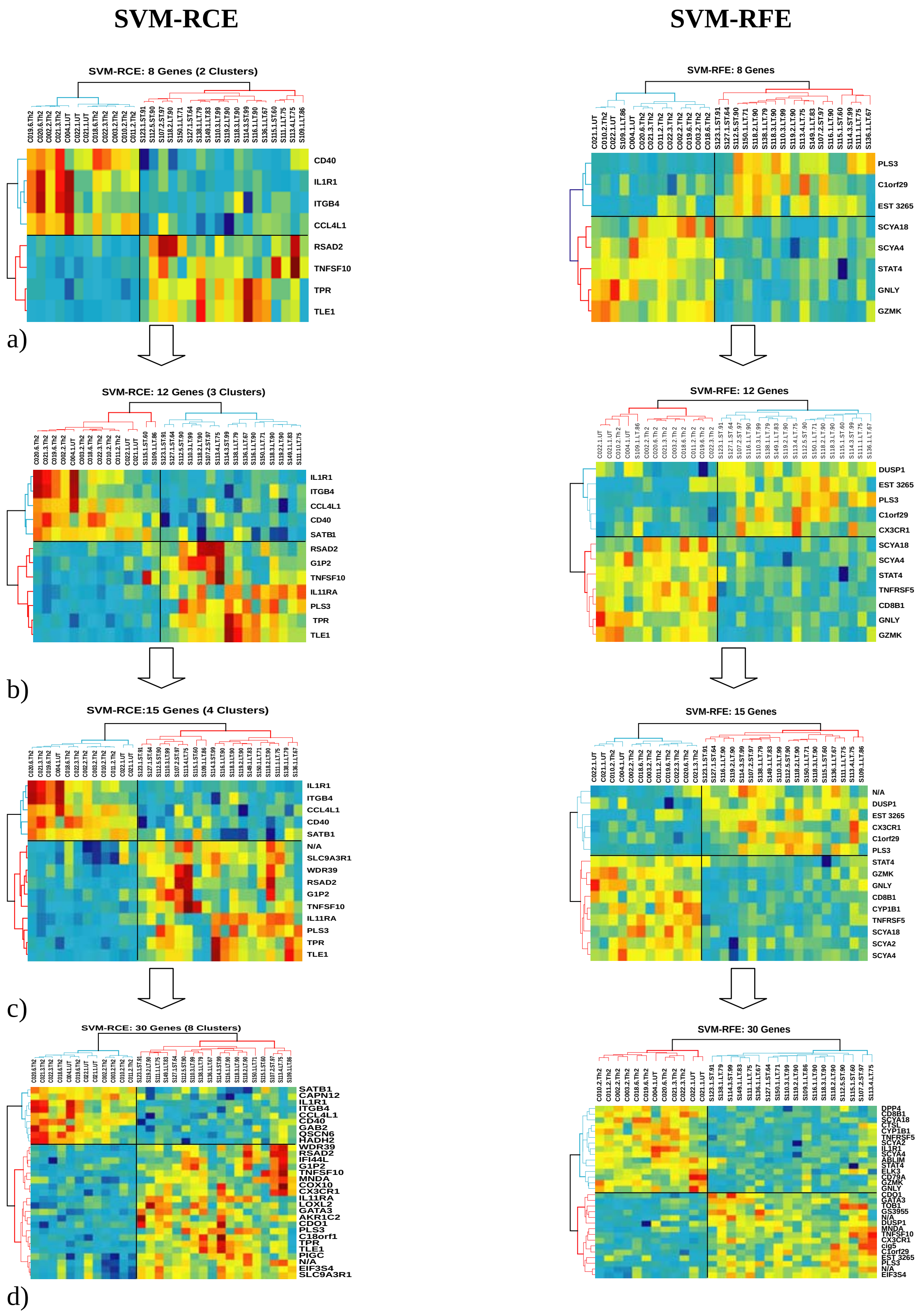

Figure 3: Left panels show an overview of the data CTCL(I) by Hierarchical clustering of different numbers of significant clusters of genes detected by SVM-RCE while the right panel show the overview of the same data with same number of genes detected by SVM-RFE. When 3 or 4 clusters are used for classification, a cluster of 4 up-regulated genes (IL-11RA, PLS3, TPR and TLE1) defines a sub-cluster of 9 or 10 patients seen in panels b and c (left). Another sub-cluster of 2 patients, S107 and S113 are characterized by extremely elevated WDR39, TNSF10, G1P2, and RSAD2. This sub-cluster structure is not seen when genes are selected by SVM-RFE, (right panels) which appears to select different genes which are more uniformly expressed across the patient samples.
